# Patient participation and learning in medical consultations about congenital heart defects

**DOI:** 10.1371/journal.pone.0220136

**Published:** 2019-07-24

**Authors:** Theres Bellander, Anna-Malin Karlsson

**Affiliations:** Department of Swedish Language and Multilingualism, Stockholm University, Stockholm, Sweden; Universiteit van Amsterdam, NETHERLANDS

## Abstract

In this article, patient activity in 8 audio recorded specialist consultations on fetal cardiology is investigated in order to explore how, why and when patients tend to participate in encounters in which the doctor dominates the interaction. The overall question is: How can the participation of patients in the consultations be connected to the development of higher levels of health literacy, i.e. to interactive literacy and to critical literacy? Patient participation is here understood as interactive action and is analyzed in terms of different interactive moves, which are related to different recurring topics. Despite the highly standardized format of the consultations, there is a large variation between the patients’ participation: between 0.7 and 2.8 moves per minute. The patients participate most during the topics ‘Prevalence’ and ‘Consultations’ and least during the topic ‘The normal heart’. Although most of the patients' moves are responses to what the doctor says, they remarkably often pose questions and use so called rejoinders. By posing questions, they take control of the information flow and sometimes even change the topics. By using rejoinders, they analyze the problems involved in the discussion e.g. by asking for clarifications or confirmation. Patients with a low over-all participation rate also use fewer moves that indicate higher literacy levels. The qualitative analysis problematizes the idea of a simple scale from basic literacy to critical literacy. Moves that indicate basic literacy skills are interactively important for the learning activity, led by the doctor. However, patients who mainly support the doctor’s initiatives don’t take the opportunity to influence the flow of information in ways that might favor their health literacy development.

## 1 Introduction

This study takes its point of departure in the discussion about patient activity in consultations with doctors, and the importance of that activity for the development of health-related knowledge. In the context of patient-centered care, the activity of the patient is considered crucial for the outcome of the care efforts. However, not all encounters between patients and doctors share the same conditions for engaging patients in analysis, choices and decision-making. In cases where knowledge is necessarily unequally distributed between doctors and patients, as is the case in genetic counselling and screening consultations, the patient needs to grasp large amounts of information before she or he can be positioned as a ‘knower’ of her or his own diagnosis and the choices she or he can make. Participation and activity here necessarily mean two different things, and their roles in building knowledge in these cases may differ from their role the typical patient-centered case.

In this article, patient activity in specialist consultations on fetal cardiology is investigated in order to explore how, why and when patients tend to participate in necessarily highly asymmetric encounters in which one speaker dominates. Qualitatively different types of participation are discussed as indicators of higher or lower levels of health literacy. The discussion also addresses how and to what extent the framework of the situation and the actions of the doctors may influence the health literacy development of the patients.

### 1.1 Health communication, health literacy and interaction

Health communication is a multidisciplinary field. Among linguists, the interaction between doctors and patients has been a favored focus of research for many decades. For linguists, i.e., conversation analysts, the medical consultation has served as a prototype for what has been called institutional interaction, i.e. conversation where institutional frameworks and roles are assumed to be enacted interactionally in systematic ways. For overviews, see [1, 2, 3]. During the most recent decades, focus in this research field has been on the asymmetries of doctor-patient interactions, where equal participation has been understood as crucial for substantial and qualitative learning to take place [4]. Later research has investigated the interactional mechanisms of patient-centered care and shared decision-making [5, 6]. In earlier research, patient participation is addressed indirectly, through a focus on how the medical professionals invite the patients to take part in key activities in the consultations. Less attention is paid to the patients’ placements of verbal contributions, both in sequences of actions and in larger consultation contexts such as discussions of diagnosis and treatments [7].

While linguistic research on health communication tends to be oriented at a micro level, examining examples of situations in great detail, more sociological research on health communication aims at understanding the relationship between different social factors and health literacy at a more general level. While early definitions of health literacy tend to focus on measurable medical knowledge in the individual [8], later approaches have been more open to including different aspects of knowledge and learning, and to focusing less on the individual’s passive reproduction of knowledge that has been acquired and more on people’s actions and practices, including their active production of appropriated knowledge [9, 10]. Within so-called New Literacy Studies, literacy is talked about in the plural, *literacies*, to emphasize that knowledge is acquired through several different practices in which people engage [11]. This study is based on the conception of health literacy found in Nutbeam [12]. Nutbeam’s three-tiered model is presented as:

- *Functional literacy*: sufficient basic skills in reading, writing and oral interaction for functioning in everyday situations.
- *Interactive literacy*: more advanced cognitive and literacy skills which together with social skills can be used to participate in everyday activities, to extract information and derive meaning from different forms of communication, and to apply new information to changing circumstances.
- *Critical literacy*: more advanced cognitive skills, which together with social skills can be applied to critically analyze information and to use this information to exert greater control over life events and situations.

Progression between the levels is not only a question of cognitive development but also of exposure and active use of different kinds of information and messages [12].

*Interactive literacy* and *critical literacy* both point towards the role of communication and the active use of language in developing health knowledge. Similar perspectives are found in the studies of Eggins, Slade and colleagues [13, 14, 15, 6, 16] where the collaborations between patients and doctors, and between health professionals, are analyzed with methods derived from systemic-functional linguistics, SFL [17, 18]. SFL describes how people use language in authentic, everyday exchanges in order to accomplish social purposes, and it offers an integrated, comprehensive and systematic model of language which enables talk to be described at different levels and in different degrees of detail. It also theorizes the links between language and social life in that it models talk as purposeful behavior and interprets it as processes of meaning making [19].

The present study makes use of an adjusted version of the model of Eggins & Slade [19], in order to quantify patients’ participation in consultations, in a way that also captures qualitative aspects that can be related to different degrees of health literacy. This model is presented in section 3.

### 1.2 Research aim and outline

The study contributes to the knowledge of the opportunities patients have to practice and develop health literacy in different contexts. More specifically, we investigate the participation of patients in specialist consultations with the aim of exploring how different kinds of participation enable learning. The overall question we seek to answer is: How can the participation of patients in the consultations be connected to the development of higher levels of health literacy, i.e. to interactive literacy and to critical literacy?

The patients in this case are pregnant women and their partners who are confronted with the suspicion that their fetus might have a severe heart defect. (We use the term *patients*, although the pregnant women and their partners are not patients in a strict sense). They participate in a diagnosis-focused and information-oriented consultation with a pediatric cardiologist. Such a case provides data which can be characterized as both universal and extreme. It is universal in that it exemplifies how people in their lives face unexpected changes that they need to make sense of and act upon. However, the situation is extreme since the information they need to incorporate is highly complex and to a large extent uncertain, while at the same time they have only a short time in which to understand the situation and make a possibly life-changing decision. According to current Swedish legislation the pregnant woman has the right to decide on termination of the pregnancy prior to a gestational age of 18 weeks and 0 days. After this period of time she will need approval from the National Board of Health and Welfare. In accordance with clinical practice, approval is not given after a gestational age of 22 weeks and 0 days [20]. In our view, the fact that the time span is concentrated and the information to be conveyed by the medical professionals is dense, puts high pressure on communication. Crucial parts of the necessary learning need to take place in the consultation room. Investigating patient participation in such conditions is particularly interesting, since similar situations can be assumed to become more common in health care, due do the development of screening technology and an increased number of treatment options (e.g. genetic counselling, cancer screening etc.).

The participation is investigated both in quantitative and qualitative terms. The quantitative analysis aims at answering the following questions:

- How much does the participation of the patients vary?
- How is the participation of the patients related to different topics?
- How is the participation of the patients distributed to different interactional functions?
- How are the interactional functions distributed in relation to different topics?

In the qualitative analysis, we focus on the consequences of different aspects of participation, as it is visible in the data, and to what extent the patients can be said to practice the different levels of health literacy. Here, the different subsections all answer the overall question: What do the patients accomplish by using different moves, especially the ones indicating interactive literacy or critical literacy? In the final section, we discuss the results and their implications for clinical practice.

## 2 Data and data collection

The study is part of the linguistic research project *Health Literacy and Knowledge Formation in the Information Society*. The project has been approved by the Local Ethical Vetting Committee in Uppsala (Lokala etikprövningsmyndigheten, Uppsala, later replaced by Etikprövningsmyndigheten) reg. no. 2015/151. The project investigates how pregnant women and their partners in Sweden experience communication with health care institutions, how they search for and value information, acquire and produce knowledge after receiving a prenatal diagnosis of a congenital heart defect in their fetus [21, 22, 23]. The project holds a large database of various types of ethnographic data, such as recordings of medical consultations before and after birth, interviews with patients and doctors, and patient blogs. In this study we examine eight audio-recorded consultations, where the patients meet a cardiologist for the first time after a suspected heart defect has been detected during a routine ultrasound screening. Due to ethical considerations, the consultations were not video recorded, but only audio recorded. However, the method for analysis used does not require visual data.

Routine ultrasound is offered to all pregnant women in Sweden at approximately 18 weeks of pregnancy and about 97% of the women consent [24]. In Sweden heart defects are the most common congenital defect and represent about 25% of all malformations in infants at birth [25]. Approximately 1,000, just below 1%, of all children born in Sweden each year are born with some kind of heart defect [26]. The detection rate of ultrasound screening is increasing and about 40% of all heart defects are discovered before birth [27]. Fetus cardiology is centralised to five hospitals in Sweden, to one of which the woman is referred after a suspicion of a heart defect has been detected [28]. In order not to disturb clinical practice more than necessary, the first-time consultation recordings were concentrated to three limited time periods: September–October 2015, February–March 2016 and September–October 2016. This means that the patient sample for this dataset was determined by the occurrences of detected heart failure during these periods. The particular dates were the result of our wish to adapt to the workload of the doctors and the nurse who provided observation and recording possibilities. The clinic is a university hospital where the staff is used to being observed for educational and research purposes. It is located in the capital area and welcomes patients from Middle and Northern Sweden, which includes both major cities and rural regions. Congenital heart failure does not seem to correlate with social, economic, ethnic or geographic factors [27, 29]. Thus, there is a good possibility of obtaining a non-biased sample by choosing time periods as the main inclusion criteria. The informants were recruited in the clinic waiting room, after the researchers had been notified by the doctors about an upcoming appointment. All patients who were approached agreed to participate. Two consultations were only observed and not audio-recorded, but documented by fieldnotes. These are not included in the present study. One of the recorded consultations was later excluded, since the couple subsequently refused participation. The present study includes all recorded consultations that were made with participant consent, thus no further selection of recorded consultations was made.

The participating couples were provided with oral and written information about the aims and methods of the project. Their consent to take part in the study was obtained before the recordings took place. (Consent from medical staff was received in advance.) We carefully considered the risk of the information and the observation itself affecting the interaction in the consultation room. In our fieldnotes, there are notations of one of the participants possibly striving at displaying “good patient” behavior in the beginning of one consultation. Otherwise, the interaction quickly became focused around the results of the examination, and the researcher in the corner was soon paid less attention.

The patients were not chosen to represent different literacy levels. One of the couples, in consultation 3, tells the researcher that they have medical training. Except for this, the participants’ levels of education or occupation are not known to the researchers. No social or geographic information was collected, except from what was occasionally revealed in the interaction.

The recordings were in a first step transcribed verbatim by two project assistants. Both assistants have a master’s degree in linguistics and are trained in conversation analysis. Both have previous experience of transcribing data for interaction analysis. In a second step all transcriptions were adjusted and refined by the project researchers, according to the conventions used by Eggins and Slade [19].

Table 1 shows an overview of the consultations. Pregnant women are shortened *Pr* and their partners *Pa*. In consultation 5 the pregnant woman's mother is present, *Prm5*. (In the result sections, the couple participating in consultation 1 is called “couple 1” etc.). Three different doctors participate, *DrA*, *DrB* and *DrC*. The same nurse, *N*, is present in six of the consultations. Four of the consultations concern relatively uncomplicated heart defects, *minor*, and four have a more complicated diagnosis, *severe*.

**Table 1:** The data: Participants, heart defect and duration of talk.

Consultation	Patients	Medical staff	Heart defect	Duration of consultation
1	Pr1, Pa1	DrB, N	Minor	12:25 min
2	Pr2, Pa2	DrC, N	Severe	19:37 min
3	Pr3, Pa3	DrA, N	Minor	26:02 min
4	Pr4, Pa4	DrB, N	Severe	33:26 min
5	Pr5, Pa5	DrB	Severe	35:40 min
6	Pr6, Pa6	DrA	Minor	46:51 min
7	Pr7, Pa7	DrA, N	Minor	50:20 min
8	Pr8, Pa8	DrA, N	Severe	51:26min
				**Average: 34:28 min**

As is shown in Table 1, the duration of the consultations does not systematically correlate with the severity of the heart defect. The shortest consultation, 1, concerns an uncomplicated diagnosis and the longest, 8, a severe one. However, the second longest, 7, is only one minute shorter than the longest, and it concerns a minor heart defect. Nor is there a correlation between the duration of the consultation and the participating doctor. Doctor A and Doctor B, who participate more than once in the data, perform both long and short consultations.

The consultation is preceded of a 15–30-minute-long ultrasound examination that takes place in silence. After this, the doctor and the patients are placed on chairs in a triangle formation around the examination bed, which serves as a table. The nurse participating in six of the eight consultations is seated most of the time at a computer in the corner of the room. She also moves in and out of the room, getting prints and taking phone calls. In each of the consultations, a researcher is present, placed outside the doctor-patient triangle.

The consultation begins with the doctor explaining the functions of a normal heart. Thereafter, she or he goes through the suspected diagnosis and possible surgery that can be performed. In parallel with the oral explanations, the Doctors A and C both used a blank piece of paper where they first drew a normal heart and after that the outlines of the malformed heart the way it appears before and would appear after surgery. Doctor B used a simple prefabricated sketch of a normal heart, where the malfunction which the possible surgery would address is drawn on the sketch during the consultation.

## 3 Method for data analysis

The project has been approved by the Local Ethical Vetting Committee in Uppsala (Lokala etikprövningsmyndigheten, Uppsala, later replaced by Etikprövningsmyndigheten), reg. no. 2015/151. Written consent was obtained by all participating patients. Oral consent was obtained by the participating doctors. In addition, the data was analyzed anonymously.

**Fig 1:**
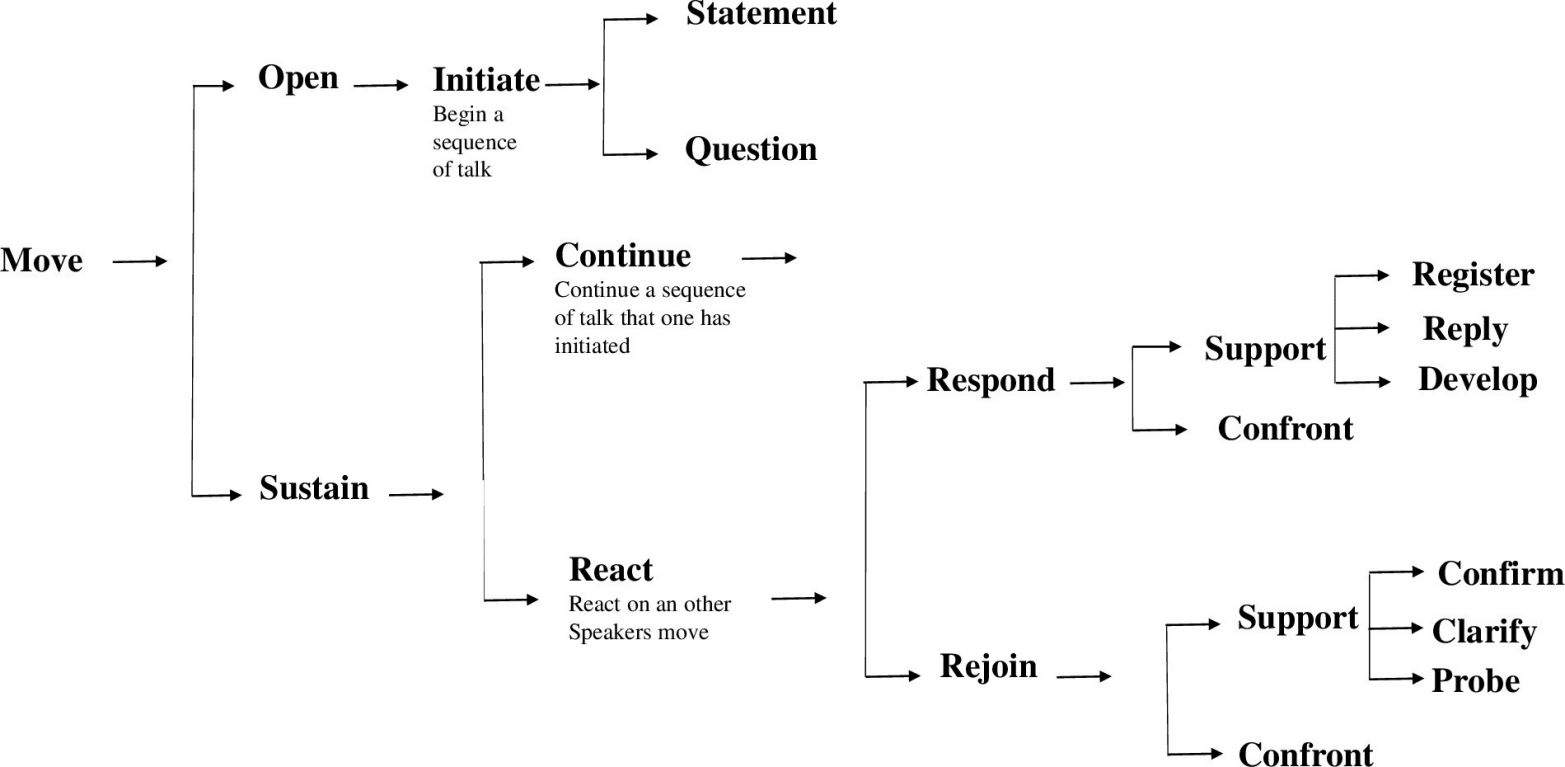
Speech function network adapted from Eggins & Slade [19].

In order to capture how and with what interactional consequences the pregnant women and their partners participate in the consultations with the cardiologist, the *turn* was chosen as the basic unit of analysis and was coded in the transcripts by the first author of the article. A turn is all the talk produced by one speaker before another speaker takes over [19]. In the next step, the first author identified and coded all the patients’ *moves*. The second author then checked the coding and ambiguous cases were carefully scrutinized by both the first and second author. Samples from the analysis were also discussed at data sessions with other researchers on two occasions. (Since the coding process was organized in order to reach consensus, which is common procedure in conversation analysis, intercoder reliability was not calculated.) *Move* was defined as the unit of discourse after which a speaker transfer could occur without it being viewed as an interruption (see [19] p. 185). Turns made by a patient most often contain one move. The moves of the patients were coded in the software *Atlas*.*ti*. (See [30] for an overview of how Atlas.ti is used for conversation analysis and discourse analysis). For the coding of moves, a speech function network inspired by Eggins & Slade [19] was used, as illustrated in Fig 1. The speech function types are sufficiently comprehensive and enable all moves to be coded.

The basic distinction in the model is the one between 1) the opening of a (new) conversation by initiating a statement or a question, and 2) sustaining earlier made moves by continuing or reacting. Each main move type can be distinguished further, through a system network, which expands towards the right in the model. Reactions are considered the most ramified. Move types representing high degrees of independent activity can be found in many places in the model. The following move types are of main interest in this study, since they can be expected to indicate and facilitate more advanced aspects of health literacy:

*Initiating moves* make it possible for the patients to start a new sequence in the consultation and thus to obtain control of the conversation by changing its focus and by developing and deepening topics. This suggests that they may indicate *interactive literacy skills* or even *critical literacy skills*.

*Prolonging moves* make it possible for the patients to add to their own previous contribution by providing further information. Prolonging moves signal the patients’ willingness to discuss. They increase the presence of dialogue and we thereby assume that they may indicate *interactive literacy skills* and *critical literacy skills*.

Based on how their interactional functions are described, we assume that also *rejoinders* may indicate interactive literacy skills or even critical literacy skills. These moves can be either *confirming*, *clarifying* or *probing*. Using confirming moves, the speaker can seek verification of what she or he has heard. With clarifying moves, the speaker can prompt additional information in order to understand a prior move. With probing moves the speaker asks the initial speaker for further details. All three types of supporting rejoinders tend to initiate sequences of talk that interrupt or postpone the speech sequence. Thus, rather than negotiating what is already on the table, rejoinders either query it–demanding further details (support)–or reject it–offering alternative explanations (reject).

A category among responding moves is *developing moves*. These build on the previous speaker’s proposition, expanding it by restating, clarifying or providing examples of what has been said. Using developing moves, the patients can slow down the flow of information provided by the doctor. This may increase the presence of dialogue and help patients to take an active part and interest in the conversation showing *interactive literacy skills*.

Other responding moves can be assumed to indicate *functional literacy skills*, since they simply mediate the patient’s participation by listening to the doctor. This is the case with *registering moves*, such as “uhum” and “okay”, but also *replies* to the doctor’s questions since replies are always expected to follow after questions in conversations. The use of registering and responding moves cannot be interpreted as a sign of the patients’ literacy level, since all conversations need moves of this kind to move forward. However, when the majority of moves fall within this latter group, it may indicate that the learning potential in that particular consultation may be lower than in consultations where a patient uses more initiating moves, prolongs, rejoinders etc.

It should be noted that the analysis of moves as indicators of different levels of literacy pays little or no respect to the quality of the moves, in terms of interactional participation or relevance in relation to the conversation as it unfolds. For instance, initiating moves can be considered as indicators of independent participation, even when the topic initiated is not relevant in the interactional context.

The patients’ participation in the consultations was measured for each conversation (and thus each participating couple) as number of moves per minute. The patient’s total amount of moves was divided by the length of the consultation.

In order to locate the moves used by patients in the content structure of the consultation, seven main organizational topics were identified and coded (1–8). To measure the time spent discussing each topic, the audio editing program *Audacity* was used.

1) ‘The normal heart’, about anatomy and functions of a normal heart
2) ‘The heart defect’, about malformations in the fetus's heart
3) ‘Treatment’, about possible surgical treatment right after birth and, in some cases, made in stages during the child’s early childhood
4) ‘Life quality’, about the child’s expected development in relation to the heart defect,
5) ‘Prevalence’, about statistics of occurrences
6) ‘Causes’, about reasons behind the heart disease
7) ‘Consultations’ about tests and further consultations in the near future
8) ‘Other’

All consultations begin with the topic 'The normal Heart' followed by 'The heart defect'. The topic ‘Consultations’ is always discussed at the end of the consultations. The order of the other topics varies. Each topic may occur several times during the consultation, i.e. the participants may return to topics they have earlier left.

## 4 Quantitative results

In this section, the results from the quantitative analysis are presented and interpreted with reference to the research questions. In the analysis of patients’ participation, their moves are measured for each consultation. This means that for each couple, literacy levels are discussed without respect to possible differences between the pregnant woman and her partner.

### 4.1 Participation and topic

How much does the participation of the patients’ participations vary? The lengths of each consultation are shown in Table 2, along with the number of moves and the results from the measurement of number of moves per minute. The average participation is 1.6 moves per minute, varying between 0.7 and 2.8. The most intensely participating couple (3) participated with nearly four times as many moves per minute as compared with the least participating couple (8).

**Table 2:** Total patient participation for each consultation.

Consultation	Length of consultation	Number of moves	Number of moves per minute
1	12:25 min	12	1.0
2	19:37 min	39	2
3	26:02 min	73	2.8
4	33:26 min	50	1.5
5	35:40 min	51	1.4
6	46:51 min	78	1.7
7	50:20 min	105	2.1
8	51:26 min	38	0.7
**Average:**	**34:28 min**	**56**	**1.6**

How is the patients’ participation related to different topics? The time spent on the different topics was measured and compared, as shown in Table 3. Most time is spent on the topic ‘Treatment’ (28% of the total consultation time), second most on the topic ‘The heart defect (23% of the time). When the number of moves made by patients is related to the different topics, it appears that the patients are most verbally active in sequences that deal with the topic ‘Prevalence’ (2.27 moves per minutes), and the topic ‘Consultations’ (2.22 moves per minute). The patients are least verbally active in sequences about ‘The normal heart’ (0.68 moves per minute). This topic is treated early in the consultations and is characterized by long explanations made by the doctor speaking alone.

**Table 3:** Total patient participation for each topic.

Topic	Minutes	Percentage of time	Number of moves	Moves per minute
The normal heart	32:33:29	12%	22	0.68
The heart defect	63:40:05	23%	99	1.6
Treatment	79:03:03	28%	113	1.43
Life quality	27:41:21	10%	37	1.35
Prevalence	06:15:05	2%	14	2.27
Causes	09:56:02	4%	14	1.46
Consultations	54:38:55	20%	121	2.22
Other	04:15:00	1%	26	6.27

Although the consultations are highly standardized, they differ as regards time spent on each topic. In Table 4, dark gray shading marks the topics that doctors and patients spend most time talking about, while a lighter shade of gray marks the topics that they spend the second most time on. In most consultations most time is spent on ‘The heart defect’, ‘Treatment’ and ‘Consultations’. In consultations 1 and 8 more percent time of the overall consultation time is spent talking about 'Life quality' than in other consultations and in consultation 5 more time is spent on the topic ‘The normal heart’.

**Table 4:** Time spent on the different topics for each couple.

	**Couple 1**		**Couple 2**		**Couple 3**		**Couple 4**	
**Topic**	**Min**	**%**	**Min**	**%**	**Min**	**%**	**Min**	**%**
The normal heart	2:18	19%	1:20	7%	4:58	19%	2:52	9%
The heart defect	3:35	29%	4:56	25%	8:50	34%	8:37	26%
Treatment	1:33	12%	3:05	16%	9:37	37%	9:51	29%
Life quality	3:02	24%	3:26	18%	0:00	0%	2:06	6%
Prevalence	0:00	0%	1:18	7%	0:00	0%	0:00	0%
Causes	0:00	0%	0:08	1%	1:59	8%	0:18	1%
Consultations	1:57	16%	5:24	28%	0:38	2%	9:42	29%
Other	0:00	0%	0:00	0%	0:00	0%	0:00	0%
In total	12:25	100%	19:37	100%	26:02	100%	33:26	100%
	**Couple 5**		**Couple 6**		**Couple 7**		**Couple 8**	
**Topic**	**Min**	**%**	**Min**	**%**	**Min**	**%**	**Min**	**%**
The normal heart	10:02	21%	4:13	8%	4:13	8%	4:15	8%
The heart defect	0:09	0%	16:24	33%	16:24	33%	16:39	32%
Treatment	21:55	47%	13:59	28%	13:59	28%	5:41	11%
Life quality	4:27	9%	2:07	19%	2:07	19%	9:42	19%
Prevalence	0:00	0%	1:47	5%	1:47	5%	2:28	5%
Causes	2:24	5%	2:00	4%	2:00	4%	1:56	4%
Consultations	6:22	14%	9:50	18%	9:50	18%	9:26	18%
Other	1:32	3%	0:00	3%	0:00	3%	1:19	3%
In total	100	100%	50:20	100%	50:20	100%	51:26	100%

The results indicate that patients are more inclined to get involved in conversation about some topics than others. The measures of number of moves per minute show higher presence of dialogue for the topics ‘Prevalence’ and ‘Consultations’ and lower for ‘The normal heart’. This indicates that the patients have more questions and find more to comment on while these topics are talked about. It is probably easier to just register the doctor’s description of ‘The normal heart’ while explanations of prevalence and future consultations might activate needs for confirmations and clarifications.

Finally, a note on the topic category ‘Other’, which applies for only two of the eight conversations, but then with a large number of moves. In both cases the topic is initiated by the patients and in both cases, it concerns issues individually related to the couple.

### 4.2 Distribution of move types

The use of different move types creates different conditions for the presence of dialogue [19]. How is the participation of the patients distributed to different move types and how are the move types distributed in relation to different topics? Since the doctor is the one providing information, it is not surprising that most of the patients’ moves are reactions to the doctors’ statements and questions. Even so, 23% of the patients’ total number of moves are initiatives in the form of questions (20%) and statements (3%). To some extent the patients also continue their initiated moves by using prolongs (7% of their total number of moves). This shows that they at least sometimes use the opportunity to try out and develop the thinking and the conclusions they make in interaction with the doctor.

Most of the patients’ moves, 49% of their total number, are responses to the doctors’ statements and questions. The most common move type used by patients is, not surprisingly, the responsive subtype registering moves (24%). Although most responses are signs of the most basic literacy skill, functional literacy, they are necessary in the consultations as the patients move the exchange towards a completion by using them. Thus, they signal that what has been said has registered and been comprehended and allow the doctor to move on (see section 5.1).

Interestingly, a fairly large number of the patients' moves consist of rejoinders (21%). They rather often respond to doctors' statements by confirming (seeking verification of what they have heard), clarifying (seeking additional information in order to understand), and probing (proposing further details or implications for confirmation by the doctor). The large number of rejoinders used shows that the patients are eager to understand the doctors’ information.

Dominating move types vary between different couples, as is shown in Table 5. Large numbers of initiatives and rejoinders indicate the presence of dialogue and interactive literacy skills. When initiated statements and questions are followed by prolongs, the presence of dialogue increases. As stated in section 3, signs of critical literacy are assumed to be found in sequences where the patients use initiatives, especially in combination with prolongs, and in sequences where they use rejoinders, especially probes.

**Table 5:** Move types for each couple.

	Couple 1		Couple 2		Couple 3		Couple 4		Couple 5		Couple 6		Couple 7		Couple 8		In total	
	Σ	%	Σ	%	Σ	%	Σ	%	Σ	%	Σ	%	Σ	%	Σ	%	Σ	%
**Initiate**	2	17%	11	28%	17	23%	15	30%	15	29%	14	18%	26	25%	5	13%	105	23%
Statement	1	8%	2	5%	2	3%	0	0%	5	10%	2	3%	2	2%	1	3%	15	3%
Question	1	8%	9	23%	15	20%	15	30%	10	20%	12	15%	24	23%	4	11%	90	20%
Prolong	0	0%	0	0%	11	15%	1	2%	5	10%	1	1%	9	9%	2	5%	29	7%
**Respond**	9	75%	22	57%	24	33%	26	52%	23	45%	48	62%	43	41%	25	66%	220	49%
Register	5	41%	8	21%	13	18%	13	26%	8	16%	29	37%	22	21%	8	21%	106	24%
Reply	1	8%	9	23%	3	4%	9	18%	5	10%	15	19%	12	11%	8	21%	62	14%
Develop	3	25%	5	13%	8	11%	4	8%	9	18%	4	5%	9	9%	9	24%	51	11%
Confront	0	0%	0	0%	0	0%	0	0%	1	2%	0	0%	0	0%	0	0%	1	0%
**Rejoinder**	1	8%	6	15%	21	29%	8	16%	8	16%	15	19%	27	25%	6	16%	93	21%
Confirm	0	0%	4	10%	5	7%	3	6%	1	2%	6	8%	8	5%	2	5%	29	7%
Clarify	1	8%	2	5%	3	4%	2	4%	3	6%	3	4%	15	3%	1	3%	30	7%
Probe	0	0%	0	0%	12	16%	3	6%	3	6%	6	8%	4	3%	3	8%	31	7%
Confront	0	0%	0	0%	1	1%	0	0%	1	2%	0	0%	0	0%	0	0%	2	0%
In total	12	100%	39	100%	73	100%	50	100%	51	100%	78	100%	105	100%	38	100%	446	100%

Table 5 shows that couples 1 and 8 stand out by using fewer initiating and prolonging moves. With consultation 1 being the shortest and 8 the longest, these two are also the consultations where patients participate with the fewest moves per minute (see Table 2). In consultations 1 and 8, the patients participate mainly by means of reactions to the doctors’ statements and questions. The number of basic registering moves is high and few initiating questions or rejoinder probes are made. In contrast, couples 3 and 7 stand out by participating with an even distribution of initiating, responding and rejoinder moves. To a fairly large extent, especially couple 3, they continue their initiatives with prolongs. They (especially couple 3) also make use of probing rejoinders and thereby promote discussion with the doctor. The patients participating in consultation 3 and 7 are also those making most moves per minute (see Table 2).

How are the patients’ contributions to the conversation related to different topics? As shown in Table 6, most initiating moves made by patients concern the topic ‘Prevalence’ (43%). Here, the patients also produce a large number of rejoinders (36%), as well as many confirming and clarifying questions. This indicates that ‘Prevalence’ is of great interest for the patients. It should be noted, though, that only 2% of the average consultation time is spent on the topic ‘Prevalence’ (see Table 3). A topic more central to the consultations is ‘The heart defect’, on which 23% of the consultation time is spent (see Table 3). For this topic, 25% of the patients’ moves are initiating moves and 35% are rejoinders (see Table 6). All rejoinder move types (confirm, clarify, probe and confront) are used in relation to this topic. Other topics where the patients produce substantial numbers of initiating and rejoinder moves are ‘Treatment’ and ‘Life quality’. When talking about the topics ‘The normal heart’ and ‘Causes’ the patients mainly contribute with responding moves.

**Table 6:** Move types in relation to the different topics.

	The normal heart		The heart defect		Treatment		Life quality		Prevalence		Causes		Consultations		Other		All topics	
	∑	%	∑	%	∑	%	∑	%	∑	%	∑	%	∑	%	∑	%	∑	%
Initiate	1	5%	25	25%	30	27%	10	27%	6	43%	2	14%	29	24%	2	8%	105	**24%**
Statement	0	0%	3	3%	4	4%	2	5%	0	0%	0	0%	5	4%	1	4%	15	3%
Question	1	5%	22	22%	26	23%	8	22%	6	43%	2	14%	24	20%	1	4%	90	20%
Prolong	0	0%	8	8%	9	8%	6	16%	0	0%	0	0%	4	3%	2	8%	29	**6**%
Respond	19	86%	32	32%	47	41%	16	43%	3	21%	9	65%	73	61%	21	80%	220	**49%**
Register	8	36%	13	13%	28	25%	6	16%	2	14%	5	36%	33	27%	11	42%	106	24%
Reply	9	41%	6	6%	7	6%	4	11%	0	0%	4	29%	26	21%	6	23%	62	14%
Develop	1	5%	13	13%	12	11%	6	16%	1	7%	0	0%	14	12%	4	15%	51	11%
Confront	1	5%	0	0%	0	0%	0	0%	0	0%	0	0%	0	0%	0	0%	1	0%
Rejoinder	2	9%	35	35%	27	24%	5	14%	5	36%	3	21%	15	12%	1	4%	93	**21%**
Confirm	1	5%	8	8%	4	4%	2	5%	3	21%	1	7%	9	8%	1	4%	29	6%
Clarify	0	0%	10	10%	12	11%	3	8%	2	14%	0	0%	3	2%	0	0%	30	7%
Probe	1	5%	15	16%	10	9%	0	0%	0	0%	2	14%	3	2%	0	0%	31	7%
Confront	0	0%	1	1%	1	1%	0	0%	0	0%	0	0%	0	0%	0	0%	2	0%
In total	22	100%	99	100%	113	100%	37	100%	14	100%	14	100%	121	100%	26	100%	446	100%

Although the doctors dominate the consultations, being the main information providers, the patients do more than just respond to the doctors’ questions and statements. To a fairly large extent, they use their slots in the conversations for initiating their own questions and statements as well as asking for confirmations and clarifications. Participating in the conversation by using few moves seems to correlate with a dominance of responding moves, while participating by using many moves yields a more even distribution of initiating, responding and rejoining moves.

## 5 Qualitative results

In this section, we discuss the functions and effects of different moves, especially the ones which are expected to indicate higher literacy levels. In the first subsection, however, we investigate the potentials in moves that indicate basic literacy levels. Here we also take a closer look at the consultations where the couples are least interactively active, to discuss what this may lead to in terms of learning. Four transcripts, one for each subsection, are provided. These have been selected from the data collection in its entirety to serve as particularly clear examples of functions and effects of different moves. The question which is answered is: What do the patients accomplish by using different moves, especially the ones indicating interactive literacy or critical literacy?

### 5.1 Acknowledging receipt

S1 Transcript (please see the full transcript 1 in S1 Transcript) shows a typical example of the patients responding to the information given by the doctor with registering moves, like “mm” and “yes”. (All transcripts are translated from Swedish.) The pregnant woman’s short registering moves signal to the doctor that she is grasping the information in a way that permits the doctor to continue the description of the malformation. This minimal acknowledging of receipt of information occurs despite the fact that it is possible for the patients to participate in more substantial ways. Several actions by the doctor provide “slots” for the patients to fill. First, the doctor is sketching while talking, which slows down the pace of the conversation and causes long pauses. This provides opportunities for the patients to ask questions or make comments with confirming, clarifying or probing rejoinders. Second, the doctor expresses uncertainty, or limited certainty, e.g. by shifting between we- and I-subjects: “*we* say that”, “considering what *we* see”, “*I* don’t think”, “*I* still haven’t been able to show”, “*I* can see”. This is known to be a strategy for involving patients and showing less authority [31, 32, 4]. Third, the doctor uses modal expressions for probability, “one *could* consider theoretically”, “this type *might* show up”, “*most often* this type of [blood] circulation *usually* works as long as it stays in the womb”, which has been described as a way to open up for questions [32, 33, 3, 4]. Fourth, the doctor uses rhetorical questions while explaining: “The question is […] what does that mean to you? What can be done?” and “Is there a threat to the fetus while it is in there?”. Although the doctor answers the questions himself or herself, the questions do leave an opening for the patients to respond with replies (see the full transcript 1 in S1 Transcript).

The doctor in many ways makes it easier for the patients to take a more active part in the conversation, but the opportunity is not taken by the patients in S1 Transcript. However, their communicative actions do keep the consultation going. In our view, the patients here display basic literacy skills, which strengthens the pupil-teacher relationship with the doctor. This may result in appropriate learning in terms of a basic understanding of the anatomy of the malfunction and of the possible surgery. However, it is not made clear if the patients “have made the knowledge their own”–an expression used by one of the doctors in an interview, as an ideal result of the consultation.

### 5.2 Slowing down the flow of information

In S2 Transcript the topic talked about is the same topic as in S1 Transcript: the heart defect. The doctor is talking while sketching on a sheet of paper (please see the full transcript 2 in S2 Transcript). In contrast to the patient in S1 Transcript, the patients here use the opportunities provided by the pauses caused by the sketching, to ask questions. Their questions function as responses to the statements in the doctor’s description. With the first question: “But does it [the blood] come up there and then it is blocked?”, the partner probes a description of the malformed heart, as perceived by him. He explores whether he has understood where the blockage is located. The pregnant woman asks a clarifying question about “these three”, which probably refers to the three arteries that lead the blood from the aorta. She asks in order to confirm that she has grasped the quick answer from the doctor by repeating the doctor’s words: “They are unaffected?”. She moves on and asks to confirm that the “blockage” is the only problem. “It is just that then? The aorta is not small?”. With their questions, the patients in S2 Transcript take control of the flow of information by slowing it down, so that they can sort out the malformations of the heart. This is achieved step by step, by probing, clarifying and confirming the information in dialogue with the doctor. The way the patients extract the information and derive meaning from it, clearly indicate high interactive literacy skills. They show ability to ask for clarification and confirmation, but also the ability to ask for information that was not provided. This means that they are able to do the same in other situations, for example on the Internet, in interaction with other patients.

### 5.3 Speeding up the process

The question asked by the patients in S3 Transcript is different, as compared with the ones asked in S2 Transcript (please see the full transcript 3 in S3 Transcript). Here the patient’s question changes the topic, and also pushes the process of the consultation forward to the phase where the nearest future is discussed. According to the template for this type of consultation, the nurse and the doctor are informing the patients about the fact that the heart defect is not caused by the actions or lifestyle of the pregnant woman. The nurse gives two examples of possible behaviors that do not affect the development of the heart: “took a glass of wine” and “carried too heavy”. She also emphasizes her statement “you should never blame yourself” by describing the impacts as “pure chance”. The doctor clarifies this by declaring that they “don’t know the reasons”. By asking a question, which is not in line with the ongoing topic, the pregnant woman signals that the information she has received on causes is enough. Thus, she takes control of the conversation and speeds up the process by initiating a new topic of her own choice: “if we chose to terminate the pregnancy how do we go about that?”. With the immediate answer, the doctor signals that she or he finds the question relevant and accepts the transition from the topic ‘Causes’ to the topic ‘Consultations’.

By asking the question, the pregnant woman shows signs of interactive literacy skills. She applies information received about causes as well as information about the heart defect and treatment discussed earlier, in order to approach the decision as to whether to terminate the pregnancy or carry to term. She signals that she has grasped the features of the heart defect and is ready to discuss options.

### 5.4 Deepening and problematizing

The sequence in S4 Transcript starts out with the doctor explaining different tests that are available and that might show chromosome abnormalities in the fetus (please see the full transcript 4 in S4 Transcript). The partner reacts with a confirming rejoinder: “We should do the amniocentesis straight off then?” With this move, he or she consolidates the doctor’ message that it is better to go for the amniocentesis right away, instead of taking and waiting for the results from a N-I-P-T (non-invasive prenatal test). In his or her next move, the partner changes the topic of the conversation from ‘Consultations’ back to the earlier topic ‘Heart defect’. By doing this he or she takes the initiative to analyze the problems in the diagnosis previously delivered by the doctor. In the grammatical form of statements, the partner uses the information he or she has received to sum up the results from the ultrasound and suggest a way to interpret the abnormalities found. The partner carries out the sequence of the analysis of the problem very carefully, introducing it with: “But if you put it in another way, one could think”. Throughout the sequence the partner uses the first person: “according to what *I* have understood”, and modal markers of probability: “it *may* even grow back”, “this *could be*”, “it *doesn’t have to* be”, “that *could* also be”. Also nouns are used to *signal* probability: “the best-case scenario” and “an option”.

In the sequence of the analysis of the problem in S4 Transcript, the partner on the one hand shows advanced cognitive skills by picking up and formulating relevant information on the diagnoses in medical terms: “this renal pelvis […] is not completely uncommon”. On the other hand, the partner shows social skills by firmly trying out his conclusions in dialogue with the doctor. Both interactive literacy and critical literacy are displayed.

## 6 Discussion

In this section, the results are discussed and the research questions are answered. We also discuss the limitations of the study, as well as possible implications for medical practice.

### 6.1 Answers and conclusions

The overall question asked in this study was: How can the participation of patients in the consultation be connected to the development of higher levels of health literacy, i.e. to interactive literacy and to critical literacy? The participation of the patients was analyzed in terms of different interactive moves, which were related to different recurring topics. We draw the following conclusions:

- The patients’ participation in the 8 consultations varied between 0.7 and 2.8 moves per minute. This means that some couples “do more things” during the consultation than others, which is not the same thing as talking more. The variation is considerable, since the consultations are highly standardized regarding topic structure, and are also relatively dominated by one speaker as compared with other doctor-patient interactions. Despite this, there seem to be opportunities for the patients to take an active part–opportunities which are not always taken.
- The patients participate more in relation to some topics and less in relation to others. Most moves per minute by the patients (2.27 and 2.22, respectively) are measured during the topics ‘Prevalence’ and ‘Consultations’. Fewest moves per minute (0.68) during ‘The normal heart’. This is a natural consequence of the unequal distribution of knowledge between doctors and patients when it comes to anatomy, and the increasing level of relevance that comes with discussing issues that to a larger extent relate to the life of the patients. Also, the patients may become more relaxed and inclined to participate as the consultation proceeds.
- Even though most of the patients' moves (49%) are responses to what the doctor says, the patients remarkably often pose questions (20%). Questions initiated by patients constitute a powerful resource for taking control of the information flow and sometimes even for changing the topics. Also, a surprisingly large number are rejoinders (21%), which is a resource for deepening and analyzing the problems involved in the discussion by asking for clarifications or confirmation, by offering further details, or by proposing implications for confirmation. Thus, the patients are not only passive receivers of information, although providing information to them is one of the main goals of the consultation.
- Most of the patients’ initiating and rejoinder moves concern the topics ‘Prevalence, ‘The heart defect’ ‘Treatment’ and Life quality’, while responses are most common during the topics ‘The normal heart’ and ‘Causes’. This indicates that the former topics are engaging the couples more than the latter, but also that some topics may allow for participation to a higher degree.
- Patients with a low over-all participation rate also show few of the moves indicating higher literacy levels.

The qualitative analysis has shown that there are problems with the idea of a simple scale from basic literacy to critical literacy. Moves expected to indicate basic literacy skills, such as registering moves, are important for the joint learning activity, led by the doctor. Thus, couples responding with these kinds of moves are not necessarily to be ascribed a low literacy level. However, they don’t take the opportunity to influence the flow of information, which might support their learning even more.

Initiating questions and rejoinders have been shown to influence the consultation in rather different ways. While initiating questions may both slow down the information flow and speed up the process by changing topic, rejoinders instead deepen the discussions of the topic at hand. In this way, rejoinders to a larger extent align with the doctor’s plan and intended flow, while questions intervene and interrupt. This may have different effects. Alignment, i.e. maintaining the topic at hand, help joining doctors and patients in a mutual project, while interventions may change the direction of the doctor’s plan, and thus interrupt possible deepening and force the joint investigation to start again, at a more general level.

In relation to this, it needs to be said that high interactivity and extensive patient participation are not always ideal from a learning perspective. Although favored by interaction-oriented research, such as [7], the idea of learning as dependent on an equal distribution of talk, quantitatively and qualitatively, needs to be analyzed for potential problems. According to our findings, variation can be found during the same consultation, and can be related to specific topics and phases in the activity. This suggests that complex health learning, especially covering issues of uncertainty, may benefit from a clear distinction between expert and patient responsibility. In our data, the doctors clearly take responsibility for grounding the description of the diagnosis in a detailed description of a normal heart. Here the patient’s participation is necessarily low and subordinated. But when shifting to future possibilities, risks and choices, the patients become more involved. This is also where the potential for developing higher levels of literacy is located.

### 6.2 Limitations

This study has focused on a specific diagnosis, with specific ethical and communicative challenges. We argue that our results have relevance for other consultation types with other patient groups, which share factors such as uncertainty in diagnosis and time pressure for crucial decision making. However, we do not claim general relevance for medical consultations at large.

A limitation of the study is that no statistical sample has been used and that no socio-demographic characteristics of the participants has been collected, and that the results thus may have limited relevance for the whole patient population. Discussions with the medical professionals, throughout the research process, have however strengthened our view that these consultations are representative and that the analysis is valid for the practice at hand. The dataset could be considered small, regarding the number of consultations as well as the number of couples and doctors participating, but it is large in relation to the detailed, qualitative analysis conducted. It should also be noted that we aim at understanding how different types of communicative action, here studied in terms of interactional moves, influence the situational learning potential in the consultations, not how different categories of patients (or doctors) act or learn.

A final limitation has to do with the audio recording, which has confined our analysis to spoken, verbal interaction. Of course, a large part of the meaning making taking place in the consultation room is embodied and visual [34]. Our findings are restricted to how the linguistic resources available to doctors and patients are used to accomplish participation.

### 6.3 Implications for practice

As medical professionals, the doctors in our study manage the consultations in order to provide as much important information as possible, so that the patients can make informed decisions. This necessarily leaves little room for the patients to take an active part in a dialogue. However, it is also clear that the doctors need evidence that the patients have received the information, showing that the patients have understood. This leads to a design where mainly registering moves are expected from the patients. If more participation is desirable, which is probably necessary for transferring responsibility for decision making to the patients, the design may need to be adjusted.

Potential for more crucial patient participation is mainly located in the parts of the consultation where treatment, prevalence and future consultations are discussed. It is our interpretation that these topics create openings for different perspectives, which makes it possible for patients to bring their views and experiences forward. Patient learning can here be supported by a joint interest in deepening the issues brought up. Thus, the doctors’ rather firm plan for the consultation does allow for occasional flexibility when needed.

Rejoinders often lead to the deepening of topics, and evoke the patients’ interactive literacy and critical literacy. Doctors could be more attentive when patients ask for confirmations and clarifications and when they inquire more details.

Patients are different from one another, both with respect to background knowledge and to interactive confidence. Coping with this by combining a highly standardized format, where “all get the same”, with compensatory tools of different kinds: e.g. rhetorical questions seems to be an appropriate strategy, not least with respect to the information-providing aim of the consultation. However, thus strategy may be less adequate for transferring responsibility for making decisions. We would once again want to point out that how the different parts of the consultation and the different topics are handled can provide different conditions for patient involvement. From starting in a medically specialized domain, with clear expert roles, the talk moves gradually over to a world of real life where the patients’ perspectives and experiences become more relevant. Often time is limited, but balancing the time spent on topics “owned by the doctor” and the time spent on topics engaging the patients may be a way to get more participation from the quieter patients.

## Supporting information

S1 Transcript Transcript 1. (DOCX)

S2 Transcript Transcript 2. (DOCX)

S3 Transcript Transcript 3. (DOCX)

S4 Transcript Transcript 4. (DOCX)
