# Supplementary material for: Patient participation and learning in medical consultations about congenital heart defects
Source: PLoS One. 2019 Jul 24;14(7):e0220136. doi: 10.1371/journal.pone.0220136 (PMC6655745; doi:10.1371/journal.pone.0220136)
Supplement: S1 Transcript — (DOCX) [file pone.0220136.s001.docx]

**Transcript 1**

| **Speaker** | **Transcription** | **Move** | **Topic** |
| --- | --- | --- | --- |
| DrB: | A third type that one could consider theoretically is extremely unusual and we say that this type might show up every twenty years or so ((pause)). The question is, considering what we see now, what does that mean to you? What can be done? |  | The heart defect |
| Pr5: | Yes | Respond, register |  |
| DrB: | Is there a threat to the fetus while it is in there? Eh, most often this type of [blood] circulation usually works as long as it stays in the womb. So I don’t think this one will succumb as a fetus. [The blood] is pumping nicely. |  |  |
| Pr5: | Mm | Respond, register |  |
| DrB: | After it is born it is important that the blood flow to the lungs is good. |  |  |
| Pr5: | Mm | Respond, register |  |
| DrB: | Then we will need sound oxygenation of the blood. |  |  |
| Pr5: | Um | Respond, register |  |
| DrB: | Uh, and I still haven’t been able to show that there is a vessel to the lungs, but it is coming, I can see that there are lung arteries |  |  |
| Pr5: | Mm | Respond, register |  |
| DrB: | I can see that there is a flow and that means that there must be a path |  |  |
| Prm5: | Yes, yes | Respond, register |  |
| DrB: | Some path out to the lung |  |  |
