# Supplementary material for: Patient participation and learning in medical consultations about congenital heart defects
Source: PLoS One. 2019 Jul 24;14(7):e0220136. doi: 10.1371/journal.pone.0220136 (PMC6655745; doi:10.1371/journal.pone.0220136)
Supplement: S2 Transcript — (DOCX) [file pone.0220136.s002.docx]

**Transcript 2**

| **Speaker** | **Transcription** | **Move** | **Topic** |
| --- | --- | --- | --- |
| DrA: | In this area there is no wall and that means the hole can be both round and oblong and lobed. It can be shaped in many ways. |  | The heart defect |
| Pa7: | But does it [the blood] come up there and then it is blocked? | Rejoinder, probe |  |
| DrA: | Yes, that’s exactly how it is, yes. |  |  |
| Pr7: | But these three, uh? | Rejoinder, clarify |  |
| DrA: | Mm, they are unaffected. |  |  |
| Pr7: | They are unaffected? | Rejoinder, confirm |  |
| DrA: | Yes |  |  |
| Pr7: | It is just that then? The aorta is not small? | Rejoinder, confirm |  |
| DrA: | No, its´s like this, it’s not abnormally small, it is a bit smaller than I had expected. |  |  |
