# Supplementary material for: Patient participation and learning in medical consultations about congenital heart defects
Source: PLoS One. 2019 Jul 24;14(7):e0220136. doi: 10.1371/journal.pone.0220136 (PMC6655745; doi:10.1371/journal.pone.0220136)
Supplement: S3 Transcript — (DOCX) [file pone.0220136.s003.docx]

**Transcript 3**

| **Speaker** | **Transcription** | **Move** | **Topic** |
| --- | --- | --- | --- |
| N: | It is important to know that you haven’t done anything wrong. This is pure chance. It is not that you took a glass of wine before you knew you were pregnant or that you carried too heavy. You should never blame yourself in this situation. |  | Causes |
| DrC: | We don´t know the reasons for this but we do know that there isn’t anything that you did or didn’t do. |  |  |
| Pr2: | No ((three second pause)) but if we chose to terminate the pregnancy how do we go about that? | Initiate, question | Consultations |
| DrC: | In that case the Fetal medicine unit will take care of you and they will tell you about the procedure. |  |  |
