# Supplementary material for: Patient participation and learning in medical consultations about congenital heart defects
Source: PLoS One. 2019 Jul 24;14(7):e0220136. doi: 10.1371/journal.pone.0220136 (PMC6655745; doi:10.1371/journal.pone.0220136)
Supplement: S4 Transcript — (DOCX) [file pone.0220136.s004.docx]

**Transcript 4**

| **Speaker** | **Transcription** | **Move** | **Topic** |
| --- | --- | --- | --- |
| DrA: | That test [N-I-P-T] is only used to decide whether to go on with the amniocentesis and we have already found an abnormality in the kidneys and a small one in the blood vessel so we have already satisfied the criteria to go on searching […] in order to find out |  | Consultations |
| Pa3 | We should do the amniocentesis straight off then? | Rejoinder, confirm |  |
| DrA: | Yes, then you should take it as soon as possible |  |  |
| Pa3: | But if you put it in another way, one could think, the best-case scenario, ((clears his throat)) that this renal pelvis, according to what I have understood it is not completely uncommon and still in pregnancy week 32 it may even grow back | Initiate, statement | The heart defect |
| DrA: | Mm |  |  |
| Pa3: | Uh, so in a best-case scenario this could be two separate incidents | Prolong |  |
| DrA: | Hm |  |  |
| Pa3: | In different places in the body by pure chance and it doesn’t have to be anything more really, that could also be the case. | Prolong |  |
| DrA: | That can also be |  |  |
| Pa3: | So that is also an option not only the worse | Prolong |  |
| DrA: | Yes, right but one has to approach to the scope of this. This is the worst case. One can always accept the mild circumstances |  |  |
| PrA: | Yes. We just want to rule out… | Prolong |  |
| DrA: | Yes. You want to know about the worst case. |  |  |
